# A Bistable Model of Cell Polarity

**DOI:** 10.1371/journal.pone.0030977

**Published:** 2012-02-23

**Authors:** Matteo Semplice, Andrea Veglio, Giovanni Naldi, Guido Serini, Andrea Gamba

**Affiliations:** 1 Department of Physics and Mathematics, Università dell'Insubria, Como, Italy; 2 Genomes and Genetics Department, Unit Physics of Biological Systems, Institut Pasteur, Paris, France; 3 Department of Mathematics “F. Enriques”, Università degli studi di Milano, Milano, Italy; 4 Laboratory of Cell Adhesion Dynamics, Institute for Cancer Research and Treatment and Department of Oncological Sciences, School of Medicine, Università degli studi di Torino, Candiolo, Italy; 5 Department of Mathematics, Politecnico di Torino, Torino, Italy; 6 Laboratory of Systems Biology, Institute for Cancer Research and Treatment, Candiolo, Italy; 7 INFN, Torino, Italy; National Cancer Center, Japan

## Abstract

Ultrasensitivity, as described by Goldbeter and Koshland, has been considered for a long time as a way to realize bistable switches in biological systems. It is not as well recognized that when ultrasensitivity and reinforcing feedback loops are present in a spatially distributed system such as the cell plasmamembrane, they may induce bistability and spatial separation of the system into distinct signaling phases. Here we suggest that bistability of ultrasensitive signaling pathways in a diffusive environment provides a basic mechanism to realize cell membrane polarity. Cell membrane polarization is a fundamental process implicated in several basic biological phenomena, such as differentiation, proliferation, migration and morphogenesis of unicellular and multicellular organisms. We describe a simple, solvable model of cell membrane polarization based on the coupling of membrane diffusion with bistable enzymatic dynamics. The model can reproduce a broad range of symmetry-breaking events, such as those observed in eukaryotic directional sensing, the apico-basal polarization of epithelium cells, the polarization of budding and mating yeast, and the formation of Ras nanoclusters in several cell types.

## Introduction

Cell polarity plays a fundamental role in cell biology. Many cellular systems use polarity not only to respond to external stimuli but also to define tissue and organ boundaries, or to proliferate. Eukariotic cells show an extraordinary ability of orienting toward sources of chemical signals through a complex mechanism of cell membrane polarization governing the early stages of chemotaxis [1]–[3]. Budding yeast undergoes polarized growth during budding and mating. Epithelial cells polarize into an apical and a basolateral region.

Cell polarization can be guided by internal or external spatial cues, such as internal landmark proteins or chemoattractant signals. Many cells polarize in order to migrate in response to external cues. For example, when presented with a gradient of chemoattractant, neutrophils, neurons, budding yeast and Dictyostelium respond with highly oriented polarity and motility towards the source of chemoattractant. This behavior is exhibited for a shallow gradient of chemoattractant. Several basic stages are required for highly oriented polarity. In fact, cells rearrange cellular components leading to the development of separate leading and trailing edges with distinct sensitivities for chemoattractant. Polarization can also occur randomly in the absence of such cues, by a spontaneous symmetry breaking mechanism [4]. For example, even when stimulated by a spatially uniform concentration of chemoattractant, neutrophils and Dictyostelium cells can break their initial symmetry, acquire distinct leading and trailing edges and start to migrate randomly [5].

Polarity corresponds to the formation of regions characterized by different concentrations of specific signaling molecules. We can consider these regions as “signaling domains” being in different “chemical phases”. A natural and general way to partition the cell plasmamembrane into regions characterized by complementary chemical phases is to couple local bistability with lateral diffusion [1], [2]. Bistability is ubiquitous in cell signaling networks, often leading to binary outcomes in response to graded stimuli [6]–[10]. The role of local bistability in clustering, and in the spatial localization of activated molecules, has however started to be appreciated only recently [1]–[3], [11]–[13].

Here we provide a simple, solvable model of cell membrane polarization based on the coupling of membrane diffusion with bistable enzymatic dynamics. Moreover, we show that the model can reproduce a broad range of symmetry-breaking events, such as those observed in eukaryotic chemotaxis, epithelial morphogenesis, and yeast polarization.

## Results

**Figure 1:**
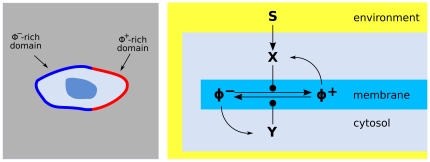
Prototypical model of cell polarization. A system of receptors 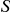 transduces an external distribution of chemotactic cues into an internal distribution of activated enzymes 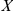, which catalyze the switch of a signaling molecule 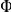 from an unactivated state 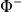 to an activated state 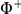. A counteracting enzyme 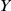 transforms the 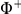 state back into 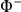. The network contains a couple of amplifying feedback loops: the signaling molecule 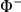 activates 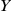 and 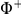 acvivates 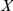. The signaling molecules 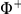, 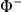 are permanently bound to the cell surface 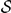 and perform diffusive motions on it, while the 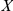, 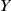 enzymes are free to shuttle between the cytosolic reservoir and the membrane. The result of the polarization process is the formation of separate domains with 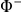-rich patches and, respectively, 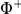-rich patches.

Our general model of chemical cell membrane polarization is an abstraction of features observed in several biological systems, where a couple of interconverting signaling molecules 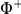, 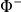 are localized on the cell plasmamembrane and are transformed into each other by a couple of counteracting enzymes 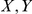 (Fig. 1). The 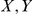 enzymes shuttle between the cytosol and the plasmamembrane, and may be activated either by a signal 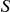 coming from the environment, or by the 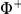, 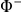 molecules themselves through local reinforcing feedback loops. The diffusivity of the 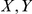 enzymes in the cytosolic reservoir is much larger than lateral mobility of molecules on the cell membrane. Therefore, an approximate equilibrium is established between the population of bound enzymes and the pool of free enzymes diffusing in the cytosolic reservoir. For instance, 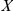 and 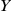 may represent a phosphatase-kinase couple that control the transition of a signaling molecule between two phosphorylation states.

In known biological models of cell membrane polarity, three-dimensional (3D) cytosolic diffusion takes place on characteristic times of the order of 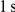, which are much faster than the characteristic times 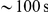 implied in two-dimensional (2D) polarization dynamics [2]. This *timescale separation* implies that the 3D distribution of cytosolic molecules can be assumed to be approximately uniform in space and slowly varying in time on the characteristic timescales of 2D polarization.

The generic microscopic picture encoded in Fig. 1 can be naturally described by means of a discrete reaction-diffusion stochastic dynamics. The dynamics is essentially confined to the cell surface since, due to timescale separation, the cytosol may be described simply as an unstructured reservoir of molecules in approximate equilibrium with the chemical factors bound to the plasmamembrane. At larger length scales, a smoother mean-field dynamics described by concentration fields emerges from the microscopic interactions of individual molecules. The mean-field dynamics can be described by an appropriate partial differential equation (PDE) model and studied with analytical and numerical methods.

Here we describe the microscopic model, derive its mean-field description, study its qualitative behavior, and compare the modeling results with available experimental data. This way, we set up a general model of chemical phase separation correctly reproducing the dynamics of cell polarization in different biological models.

### Microscopic model

Membrane polarization is a spatially distributed process characterized by stochasticity, excitability [14], and the coupling of the 2D dynamics of membrane-bound molecules with the 3D cytosolic dynamics. The process can be conveniently described by using a lattice approximation, *i.e.* by representing the cell membrane as a 2D lattice with sites populated by discrete amounts of molecules, while reactions and diffusive jumps are realized as stochastic processes according to the rules of chemical kinetics. The coupling to the cytosol is described by allowing shuttlying of molecules between the 2D lattice and an unstructured reservoir representing the 3D cytosolic volume. From the stochastic process we then derive a macroscopic *mean field* model, where populations of molecules are described by continuous density fields, and their stochastic fluctuations are encoded into effective “noise” terms [12].

Each site 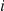 of the 2D lattice is populated by a discrete number of molecules of the relevant chemical factors. The probability distribution 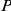 of the molecule population evolves in time according to standard master equations taking into account all possible chemical conversions and diffusion jumps [15]. For instance, the process of conversion between 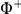 and 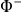 signaling molecules on the 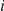-th lattice site is described by the following terms of the master equation (see Fig. 1):
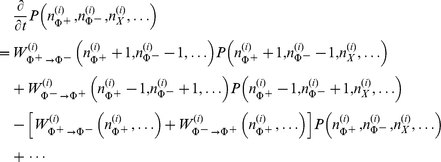
where 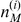 is the number of molecules of type 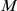 on lattice site 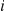, and 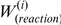 is the transition rate for the given *reaction* on site 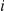. Moreover, we assume that a pool of 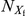, 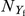 free molecules 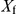, 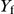 populates the cytosol. A complete list of reaction and transition rates of the signaling network described in Fig. 1 is given in Table 1. 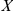 molecules activated by the external signal 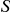 via receptors are denoted by the symbol 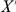, while 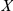 molecules activated via the feedback loop are denoted by the symbol 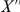 (Fig. 1). Diffusion of the 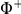 and 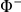 molecules on the cell membrane surface is represented by jumps from a site 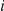 to a neighboring site 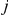 with rate 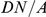, where 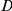 is the diffusivity, 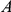 is cell membrane area, and 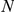 is the number of lattice sites. Diffusion of the 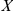 and 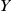 enzymes on the cell membrane is neglected. Enzymatic reaction rates are approximated by Michaelis-Menten terms.

**Table 1:** Reactions belonging to the signaling pathway of Fig. 1 and corresponding transition rates 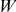.

Reaction	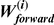	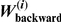
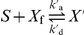	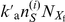	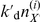
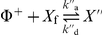	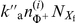	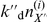
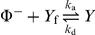	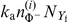	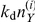
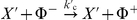	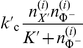	
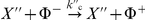	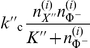	
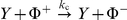	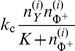	

We denote by 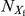 and 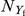 the number of free 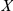, 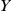 molecules available in the cytosolic reservoir, by 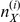 the number of 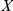 molecules on site 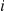 that are activated by the external signal 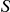 via receptors, by 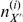 the number of 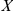 molecules on site 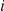 that are activated via the feedback loop in Fig. 1.

### Mean-field model

In the mean-field approximation, molecule distributions are described by continuous concentration fields, and stochasticity is encoded into an effective noise term [15]. Concentration fields are approximations to averages of molecule numbers over small neighborhoods 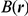 of points 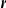 on the cell membrane surface:
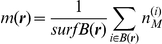
and it is assumed that the size of the neighborhood is larger than the typical molecule size, but much smaller than the typical size of the cell. Low occupation numbers 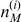 correspond to higher relative fluctuations around the mean-field concentration 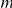.

From the spatially distributed signaling pathway of Fig. 1, Table 1, we derive the following mean-field equations:
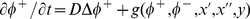
(1)

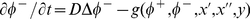
(2)

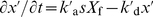
(3)

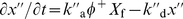
(4)

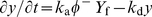
(5)

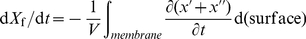
(6)

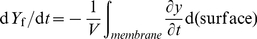
(7)where 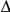 is a Laplace-Beltrami operator [16] representing diffusion on the cell surface, 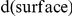 is the area element on the cell membrane surface 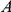, 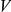 is the cell volume, and
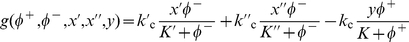
(8)describes the enzymatic conversion of 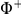 and 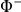.

Thermal and chemical reaction noise can be taken into account by adding the corresponding randomly fluctuating terms in the right hand side of (1–7) [15].

### Local equilibria

At equilibrium, the distribution of membrane-bound enzymes is “slaved” to the surface distribution of receptors and of 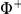, 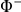 molecules (*cf.* 3–5):
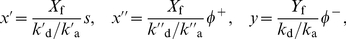
(9)while the amount of free cytosolic enzymes is a decreasing function of the *total* numbers of activated receptors and 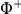, 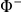 molecules (*cf.* 3–7):
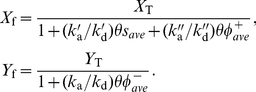
(10)Here 
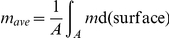
 denotes the average of the molecule distribution 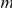 over the cell membrane surface 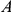, and 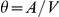 is the factor needed to convert surface concentrations (measured in 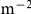) into volume concentrations (measured in 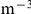).

Finally, the total amount of 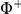 and 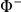 is conserved (*cf.* 1–2), then
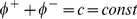
(11)


### Effective potential

Timescale separation implies that the equilibria (9, 10) for 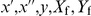 are reached in much faster time than the equilibria for the surface distributions 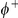, 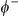 of signaling molecules. This fact suggests a convenient way to study the dynamic of cell membrane polarization, namely to substitute the fast variables 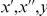 in equations (1, 2) with their steady state expressions (9, 10). The rationale here is that the concentrations (9, 10) are approximately stationary on timescales which are much shorter than the typical timescales of 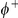, 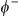 variation, and slowly vary on longer timescales. This procedure coincides with the *quasi steady state approximation* used for instance in the derivation of Michaelis-Menten laws from the theory of the transition state [17].

**Figure 2:**
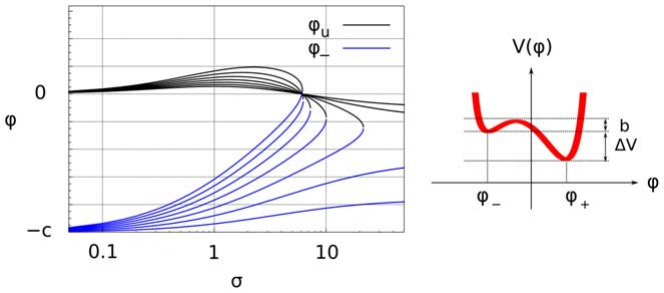
Stable chemical phases. Left: relative concentrations of signaling molecules in the stable chemical phase 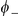 and unstable chemical phase 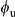, as a function of the renormalized activation signal 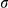 (19) (black) and for different values of the saturation constant 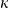. Right: Behavior of the potential 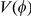, as a function of the phase 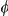, see (13). The potential 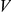 has two minima: the left-hand one corresponding to a stable 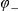-rich and the right-hand one corresponding to a stable 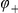-rich phase. The two phases are separated by an effective energy barrier. The existence of the two distinct stable chemical phases is called bistability.

By using the conservation law (11) we are finally reduced to consider the dynamics of *a single relevant degree of freedom*

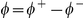
which obeys the dynamic equation
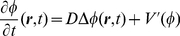
(12)with:
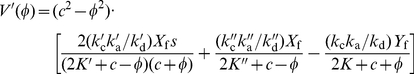
(13)(see Fig. 2). In what follows, we will assume for simplicity 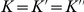. For slowly varying 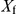, 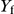, equation (12) can be written in the variational form [18]:
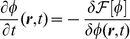
(14)with
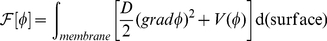
(15)showing that the dynamics of the signaling field 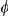 tends to minimize 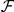, which plays the role of an *effective energy functional*.

It is worth observing here that 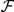 does not measure the actual energy consumed by chemical reactions, but is just a convenient mathematical bookkeeping tool which allows to determine the direction of catalytic and diffusion processes taking place in any given point on the membrane surface, at any given instant of time.

Solutions of equation (12) are obtained from
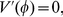
(16)which, once solved, gives two stable equilibrium values 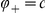, 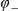 and one unstable equilibrium 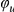. The values 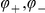 correspond to distinct, stable, uniform chemical phases, enriched respectively in the signaling molecules 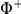 and 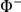. We refer to the existence of two distinct stable chemical phases as *bistability*. The explicit concentration values are
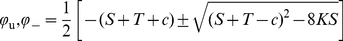
(17)where
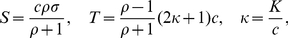
(18)with
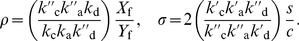
(19)Eqs. (17–19) show that the concentration values (17) are completely controlled by the *enzyme ratio* 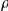, which measures the relative strength of the counteracting 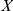 and 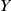 enzimes, and by the *renormalized activation signal* 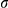.

A graph of the concentration values in the two stable phases is given in Fig. 2. An important consequence of the existence of two distinct, locally stable phases is that different regions of the cell membrane can be occupied by different phases, giving rise to patterning into distinct *signaling domains*.

**Figure 3:**
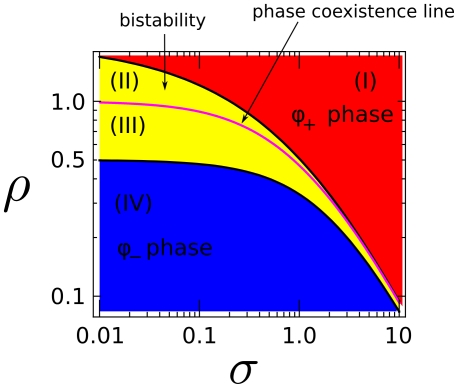
Bistability region, yellow region (II)-(III), as a function of the level of external renormalized stimulation 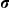
 **for** 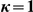 **.** The purple line corresponds to phase coexistence (polarization) and is an attractor for the polarization dynamics. The two stable domains, blue (IV) and red (I), correspond to the two 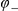 and 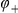 stable phases.

Patterning is possible only if the enzyme ratio 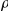 lies in the *bistability region* shown in Fig. 3 (see Supplementary Material Text S1). The enzyme ratio 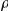 may therefore also be called a *bistability parameter* for the pathway of Fig. 1.

### Polarity

The cell membrane is *polarized* when it is divided into two complementary regions, stably occupied by one of two distinct chemical phases, separated by a thin diffusive interface. Stable polarized equilibria are reached when the effective energy 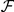 is minimal, *i.e.* when both terms in Eq. (15) take on their minimal values. If *e.g.* 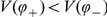, no polarized configuration can be stable because the energy can still decrease by extending the area covered by the 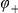 phase, which has lower energy than the 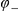 phase. The same is true if 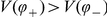. Therefore, stability of polarized equilibria (or *phase coexistence*) is possible only if the following mathematical condition is satisfied:
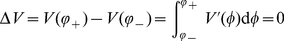
(20)Equations (13, 20) together provide an integral equation for the value at equilibrium of the enzyme ratio 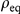 (see Supplementary Material Text S1), that can be solved numerically to determine the *phase coexistence line* 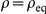 (Fig. 3).

The second condition for energy minimization is that the square gradient term in Eq. (15) is minimized. The main contribution from this term comes from the interface between regions occupied by uniform distributions of the 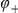 and 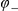 phase: energy minimization implies therefore minimization of the length of the interface that separates the two phases. The minimal value for the interface length is obtained when the cell membrane is polarized in two complementary caps, separated by a circular interface.

**Figure 4:**
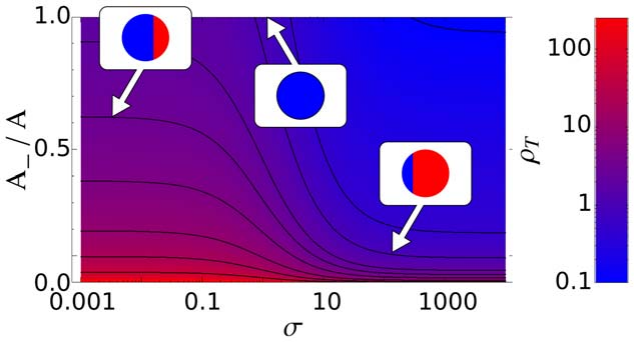
In the equilibrium state the circular patches occupied by the 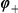
 **and** 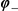 **phases have areas, respectively,** 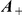 **and** 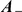 **.** Here we show the ratio 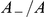 at different values of the stimulation 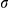. Curves are plotted from top to bottom with increasing ratio of the initial enzymes quantities 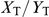. Each curve shows two plateaux that are approximatively independent of the signal 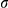. For small 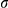 the system is dominated by the mutual interaction between 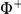 and 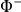, i.e., by the feedback loop, whilst for large 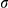 the system is dominated by the interaction with receptors, i.e., by the external signal.

In the equilibrium state 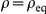, the circular patches occupied by the 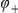 and 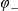 phases have areas 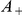 and 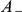 determined by the integral constraints (10). The two areas can be explicity computed if the size of the interfacial region separating the two patches is negligible with respect to the cell size (Fig. 4 and Supplementary Material Text S1). For small values of the stimulation 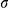, patches of the 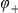 and 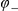 phase are mainly sustained by positive feedback loops, while for large values of 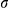, they are mainly sustained by receptor activity. The two regimes correspond to the two asymptotic plateaux appearing in Fig. 4, respectively for small and large values of the stimulation 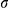. It is worth observing here that on two plateaux the areas of the signaling patches are almost *insensitive* to the absolute value of the external stimulation 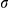, in agreement with experimental observations ([19], Fig. 3).

### Nucleation of signaling patches

The evolution from a quiescent state towards polarized equilibria involves a complex dynamics of nucleation and competitive growth of signaling patches. Similar processes have been known for a long time in the physics of materials [20], [21].

Let us assume that in the quiescent state, the plasmamembrane is uniformly occupied by the 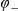 phase (Fig. 3, region II). By switching on a strong enough external stimulation field at initial time, the plasmamembrane can be brought out of chemical equilibrium, making the 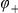 phase energetically more favorable than the 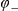 phase. The energetic barrier 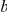 between the two potential wells (see Fig. 2) blocks however the continuous transition of the 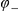 phase into the 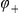 phase. The transition can take place only by the formation of sizable initial germs of the 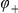 phase, driven by random thermal and/or chemical processes. Actually, small germs are mainly destroyed by diffusion, while germs larger than a critical size 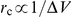 expand in the 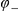 sea with a front velocity 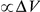 [1], [20], [22]. The larger the barrier 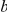, the longer the waiting time for the first appearance of a sizable germ of the 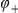 phase.

Once the first sizable germ appear, the transition towards the 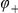 phase is initially limited only by the velocity of front propagation 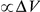. However, the growth of the 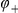 phase implies depletion of the cytosolic 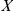 population, repletion of the cytosolic 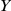 population, and decrease of 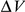, *cf.* equations (10, 13, 19). Thus, the process of growth of the 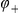 phase slows down as time advances. The cytosolic reservoir of 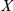 and 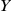 enzymes works here as a negative feedback control that drives the plasmamembrane towards the phase coexistence line (Fig. 3) and makes polarization possible.

**Figure 5:**
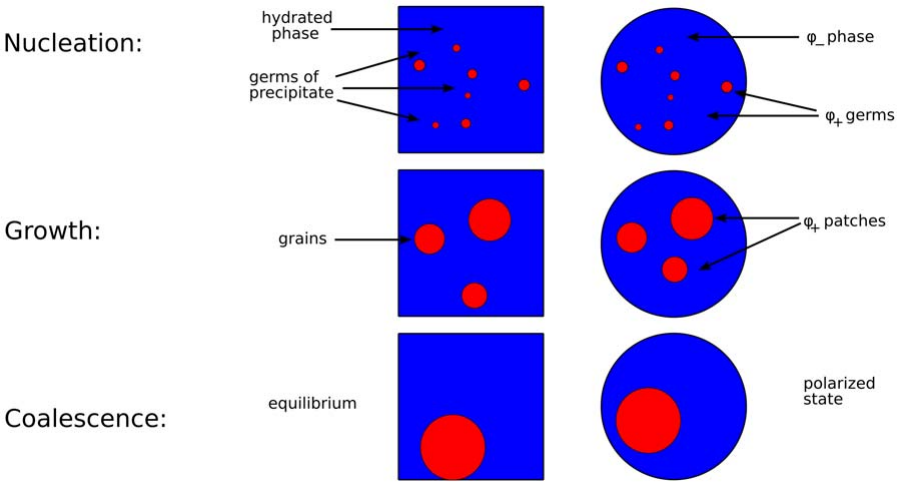
Physical analogy: membrane polarization and precipitation from a supersaturated solution. At initial time, the concentration of some molecule 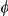 is higher than the critical value 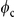, so that a small fluctuation, or an impurity, can easily give rise to the formation of small germs of precipitate. Germs larger than a critical size 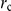 grow steadily, while germs smaller than 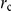 are dissolved by diffusion. As the size of the germs grows, the molecule 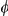 is extracted from the hydrated phase and transferred to the solid phase, moving the concentration 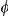 closer to the critical value 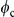, increasing the value of 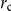, and correspondingly slowing down the process of germ growth. Grains that were initially larger than 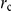 are dissolved, so that larger grains grow at the expense of the smaller grains. Eventually, an equilibrium is reached when 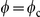 and a single large grain of precipitate survives.

As soon as the plasmamembrane is driven towards the phase coexistence line, the potential difference 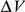 decreases and the critical radius 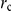 gets larger, so that patches that were previously growing fall below the critical size 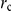 and start shrinking. Thus, large patches grow at the expense of smaller patches until a single patch survives. This kind of competitive growth of patches has been known for a long time in the physics of materials as *Lifshitz-Slyozov coarsening* [2], [3], [20], [21]. The corresponding dynamics may be understood via a simple physical analogy with the nonequilibrium process taking place during the formation of precipitate from a supersaturated solution (see Fig. 5). At initial time, the concentration of some molecule 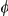 is higher than the critical value 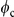, so that a small fluctuation, or an impurity, can easily give rise to the formation of small germs of precipitate. Germs larger than a critical size 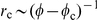 grow steadily, while germs smaller than 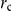 are dissolved by diffusion. As the size of the germs grows, the molecule 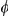 is extracted from the hydrated phase and transferred to the solid phase, moving the concentration 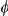 closer to the critical value 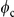, increasing the value of 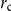, and correspondingly slowing down the process of germ growth. Grains that were initially larger than 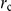 are dissolved, so that larger grains grow at the expense of the smaller grains. Eventually, an equilibrium is reached when 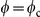 and a single large grain of precipitate survives.

### Robustness

An important result of the mathematical theory of phase ordering processes [20] guarantees that the scenario described in the previous paragraph does not depend on details such as the precise values of reaction and diffusion rate constants, on the identity of the individual biochemical factors, or on the precise analytical expressions derived from the law of mass action, but only on the following set of *robust properties* of the signaling network [2], [3], [20].

1. *Single relevant concentration field:* The polarization state of the cell membrane can be described in terms of a single relevant concentration field 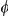, while the values of other concentration fields can be derived by approximate equilibrium relations. The evolution equation for 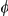 can be expressed in terms of an effective energy potential 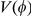.
2. *Bistability:* Feedback loops embedded in the signaling network allow for the realization of distinct, locally stable chemical phases, separated by a potential energy difference 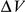.
3. *Self-tuning:* A global feedback mechanism drives the energy difference 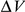 to zero, thus bringing the plasmamembrane towards the coexistence of the two chemical phases.

The robustness of our approach has important implications.

First of all, it suggests that polarization phenomena observed in distant biological models can be described mathematically in a unified way since they share a set of common features.

Secondly, it implies that robust quantitative results can be independent on the detailed knowledge of a majority of microscopic details. This property cannot be underestimated in the study of biological phenomena where the relative abundances of biochemical factors, their interactions and reaction rates are often known with comparatively poor accuracy.

### Eukariotic chemotaxis

Experiments with Dictyostelium cells exposed to uniform concentrations of cyclic AMP (cAMP) reveal a complex dynamics of membrane polarization into signaling domains enriched in either phosphatidinositol bisphosphate (PIP2) or trisphosphate (PIP3) [19]. Two enzymes, phosphatidylinositol 3-kinase (PI3K) and phosphatase and tensin homolog (PTEN), respectively, transform PIP2 into PIP3 and vice versa. The phospholipids are permanently bound to the inner face of the cell membrane, while PI3K and PTEN diffuse in the cell volume and are active only when they are adsorbed by the membrane. PI3K adsorption takes place through binding to receptors activated by the extracellular attractant signal. This way, the external attractant field is coupled to the inner dynamic of the cell. PTEN adsorption takes place through binding to the PTEN product, PIP2. This process introduces a positive feedback loop in the system dynamics [19].

In experiments, cells initially at rest are exposed to a sudden increase in the concentration of uniformly distributed extracellular signal and allowed to relax to equilibrium [19], [23]. During this interval of time a complex relaxational dynamics towards the final polarized state is observed. PIP3 patches are visualized by the use of fluorescent PH-Crac, a molecule that binds to a PH-binding domain present on the PIP3 molecule. The increase in PIP3 in the plasmamembrane signaling domain is accompanied by a corresponding decrease of PH-Crac from the cytosol: the decrease in cytosolic PH-Crac fluorescence is therefore a measure of the total amount of PIP3 in the membrane. A puzzlying aspect here is that plasmamembrane polarization seems to take place in two distinct stages. The initial stimulation with cAMP induces a uniform but transient increase in plasmamembrane PIP3 levels, of the duration of approximately 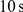. A second increase in PIP3 levels takes place after 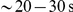, but is now localized in isolated, fluctuating domains, that occupy only a fraction of the membrane surface.

The decay of the initial uniform PIP3 burst suggests that an adaptation mechanism is at work [24]. The origin of the adaptation is likely upstream of PI3K [25]. However, the origin of the subsequent birth of localized PIP3 spots remains unclear. During the whole process PTEN and PI3K colocalize with their products, respectively PIP2 and PIP3 [19]. Although the appearence of PIP3 patches is triggered by cAMP, their size is approximately independent on cAMP levels over a wide range of concentrations, suggesting that the patches are self-organizing structures triggered by cAMP [19]. PIP3 patches show a competitive growth dynamics, with large clusters growing at the expense of smaller ones.

Colocalization of enzymes with their products implies the existence of positive feedbacks involving PIP2 and PTEN, as well as PIP3 and PI3K. Biochemical data confirm the existence of a PIP2–PTEN positive feedback loop, due to a PIP2-binding domain of PTEN [26]–[29], and of a PIP3–PI3K positive feedback loop at least in part mediated by actin [24], [30]–[32].

**Figure 6:**
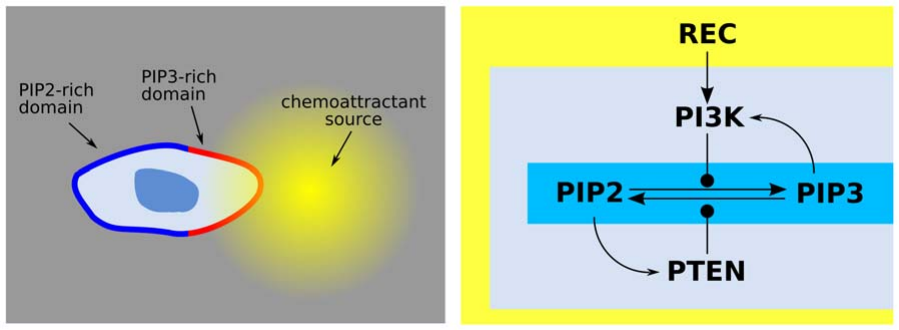
Model of chemotactic polarization. With respect to the abstract scheme in Fig. 1 we have the identification 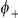 = PIP3, 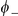 = PIP2, 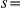
REC. The PIP3-rich domain corresponds to the presence of a high concentration of chemoattractant factor.

The structure of the PIP2–PIP3 signaling network has the form described in Fig. 6, which fits with the abstract scheme (1–8, Fig. 1) once we identify 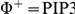, 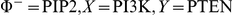.

**Figure 7:**
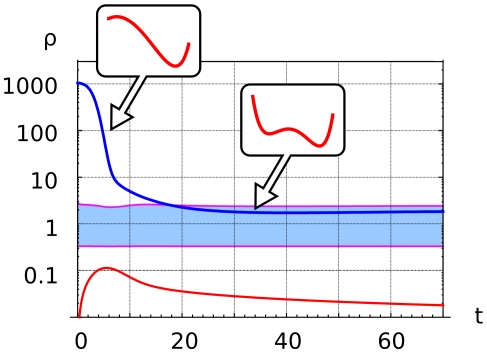
We simulated a spatially homogeneous version of the Model. We mimick the experimental conditions by switching on receptor activation at initial time. The phospholipid concentration field is driven by the slow variation in time of the effective potential 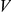, that follows the slow variation of the enzyme ratio 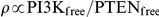. Receptor activation at 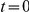 (blue line) induces a uniform increase of PI3K, PIP3 on the whole plasmamembrane, which corresponds to the appearance of a single potential well centered in the PIP3-rich region. The enzyme ratio 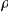 decreases, corresponding to PI3K recruitment to the plasmamembrane and PTEN relocation to the cytosol (blue line). When the enzyme ratio crosses the boundary of the bistable region (light blue area) the effective potential 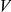 develops a secondary potential well centered in the PIP2-rich region.

To understand the origin of the two-stage plasmamembran e polarization dynamics we start by simulating a spatially homogeneous version of Model (1–8). We mimick the experimental conditions by switching on receptor activation at initial time. To take into account the initial transient adaptation we let the input signal 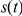 adapt in the experimentally observed time 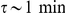 (Fig. 7). As discussed above, the evolution of the phospholipid concentration field is driven by the slow variation in time of the effective potential 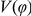, that follows the slow variation of the enzyme ratio 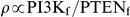 (13, 19).

Receptor activation at 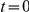 (Fig. 7, red line) induces a uniform increase of PI3K–PIP3 on the whole plasmamembrane, which corresponds to the appearance of a single potential well centered in the PIP3-rich region. The PI3K/PTEN enzyme ratio 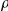 decreases, corresponding to PI3K recruitment to the plasmamembrane and PTEN relocation to the cytosol (Fig. 7, red line). When the enzyme ratio crosses the boundary of the bistable region (Fig. 7, blue curve) the effective potential 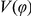 develops a secondary potential well centered in the PIP2-rich region.

The appearence of a secondary potential well follows from the increased concentration of cytosolic PTEN, which stabilizes the PIP2-rich phase. The potential barrier between the PIP2- and the PIP3-rich phase blocks further *uniform* increase in the PIP3 levels, but still allows the formation of locally enriched PIP3-rich regions through a nucleation and growth dynamics, as described above.

**Figure 8:**
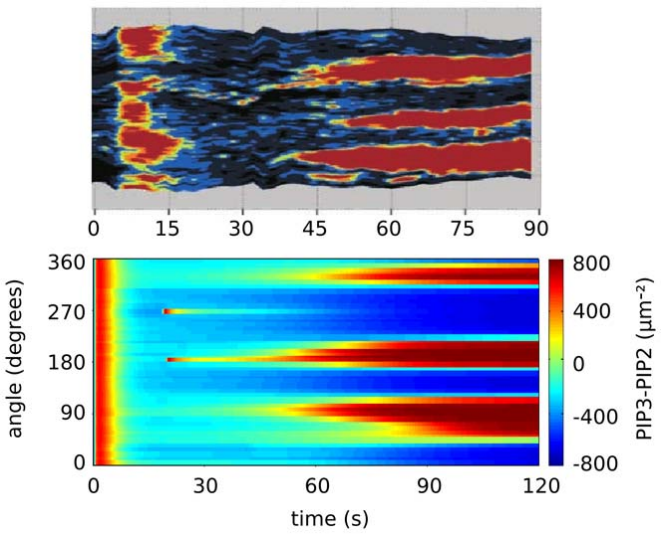
Kimograph for a simulation of the full, spatially distributed, chemotaxis system. In the simulation, before starting to stimulate cells with a uniform concentration of cAMP, the system is left to relax with zero signal until the levels of the relevant factors become stationary. Then, the stimulation is switched on at time 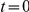, when we also impose a gaussian noise on the uniform concentration background in order to mimick random inhomogeneities. We compare the experimental results reported in Reference [19] with the simulations of model (1–8). The kimograph shows the time evolution of simulated PIP3 levels along the major cell perimeter. Time 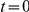 in the simulation is to be compared with time 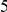 s in the experiment.

In order to validate the present scenario we have simulated the full, spatially distributed system (1–8) by using a finite-element method, with 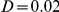 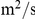 and other parameters values reported from the literature (Table 2). Thermal and chemical reaction noise is taken into account by adding an additive random perturbation in the r.h.s. of (1, 2) (see Methods). Noise has the effect of creating germs of the PIP3-rich phase as localized, rare concentration fluctuations. In the simulation, before starting to stimulate cells with a uniform concentration of cAMP, the system is left to relax with zero signal until the levels of the relevant factors become stationary and the cell membrane becomes uniformly covered by the PIP2-rich phase (blue, Fig. 8b). The stimulation is switched on at time 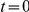, when we also impose a 5% Gaussian noise on the uniform concentration background in order to mimick random inhomogeneities. In Fig. 8 we compare the experimental results reported in Ref. [19] with the simulations of model (1–8).

**Table 2:** Parameters used in the simulations of Eukariotic chemotaxis (Dictyostelium cells).

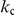	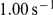	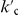	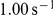	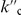	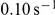	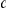	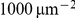
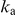	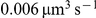	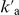	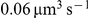	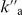	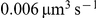	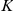	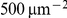
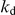	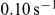	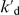	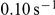	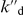	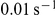	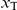	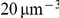
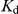	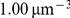	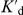	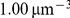	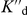	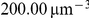	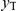	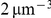

We have simulated the full, spatially distributed system (1–8) by using a Finite Element Method, with the present parameter values, that were extracted from the literature.

In both experiments (Fig. 8a) and simulations (Fig. 8b), switching on receptor activation leads to a transient increase in PIP3 concentrations (Fig. 8a). After a characteristic time of 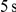, PIP3 levels decrease by adaptation. After 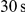 new PIP3 patches are nucleated and grow.

**Figure 9:**
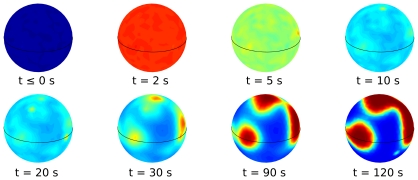
The dynamics of the simulated 3D spatially distributed model for different times. The colorbar is the same as in Fig. 9, the major cell perimeter is the one considered in Fig. 9.

The kimograph in Fig. 8b shows the time evolution of simulated PIP3 levels along the major cell perimeter, while Fig. 9 shows this very same dynamics in 3D. Similarly to what observed in experiments, a transient, uniform increase in PIP3 levels is followed by a second regime where localized PIP3 patches phase nucleate and grow competitively in a PIP2-rich sea. In both the experiments and simulations, the speed of patch growth slows down with time. The features of the simulated dynamics are therefore completely consistent with the experimental data.

Simulation data reported in Fig. 9 hint at the 3D patch dynamics that we expect will be observed when dynamic 3D reconstructions of PIP3 patches in chemotactic experiments will become available.

### Epithelial polarization

**Figure 10:**
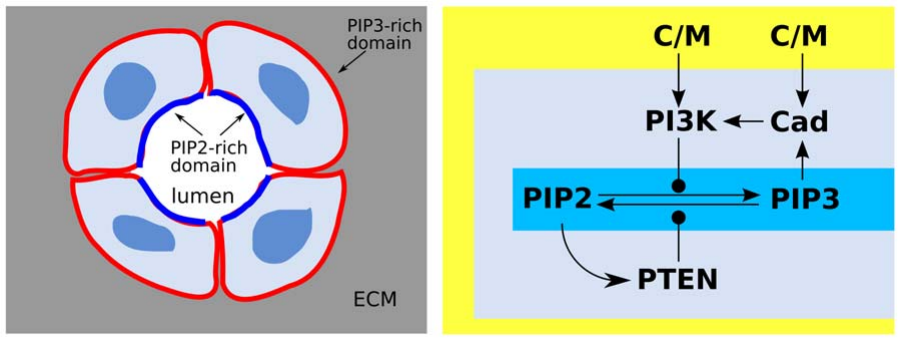
Model of epithelial polarization, with respect to the scheme in **Fig. 1** we identify 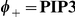
 **,** 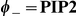 **,** 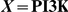 **,** 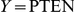 **, and** 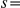 **C/M.** To bind PI3K, cadherins must be activated by engagement with cadherins of a neighboring cell. The PIP2, PIP3 localization is central in the establishment of epithelial apico-basal orientation.

In multicellular organisms, epithelial cells form layers separating compartments responsible for different physiological functions. At the early stage of epithelial layer formation, each cell of an aggregate defines a basal and an apical side. The *in vivo* process of epithelial morphogenesis is recapitulated in well established *in vitro* cell systems [33]: canine kidney cells are seeded in three-dimensional gels, where they divide and form cysts, *i.e.* hollow multicellular aggregates [33]. PIP2, PIP3 localization is central in the establishment of epithelial apico-basal polarity [34]. The apical side facing the cyst lumen is characterized by high PTEN–PIP2 plasmamembrane levels, while the basolateral side is characterized by high PI3K–PIP3 levels (Fig. 10a). PTEN levels at the plasmamembrane are controlled by its binding to PIP2, thus realizing a positive feedback loop (Fig. 10b). PI3K levels in the membrane are controlled by its binding to cell-cell adhesive receptors, cadherins, and cell-matrix adhesive receptors integrins. To bind PI3K, cadherins must be activated by engagement with cadherins of a neighboring cell (C/M in Fig. 10b) [35]. PI3K is activated when associated with either activated cadherins or integrins. Since PIP3 stabilizes the activated form Cad [36], these interactions create a positive PI3K–PIP3 feedback loop, mediated by the existence of cell/cell contacts (Fig. 10).

Before polarization, cadherins and integrins are activated along the whole plasmamembrane and PIP3 uniformly prevails on PIP2 determining a stable PIP3-rich phase over the whole membrane. A local depletion of PI3K–PIP3 can be created if a large enough membrane area with disrupted cell-cell links is formed [37], thereby breaking the PI3K–PIP3 feedback loop (Fig. 6) and originating a germ of a PIP2-rich phase (Figs. 1b and 2). The creation of this initial germ takes place by active transport of vesicles enriched in PTEN–PIP2 and antiadhesive factors to the midpoint of the mitotic spindle during the process of cell division [38], [39]. After the formation of the initial PTEN–PIP2-rich germ, the PTEN–PIP2 feedback loop may locally prevail, inducing a PIP2 and PIP3 surface compartmentalization that splits the cell membrane in two regions, characterized by different chemical concentrations of the signaling molecules.

The structure of the PIP2–PIP3 signaling network in epithelial polarization has the form described in Fig. 10, which fits with the abstract scheme (1–8, Fig. 1) once we identify 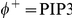, 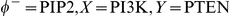. These are the same identification we used in the study of chemotactic polarization, but now with interactions and parameter values characteristic for epithelial polarity.

We have simulated Model (1–8) with parameter values compatible with the interactions described in Fig. 10 for the process of epithelial polarization (Table 3). At initial time the plasmamembrane is in a uniform PIP3-rich state. We than create a circular patch of radius 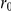 of the PIP2-rich phase of radius 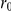 and investigate its dynamics to check whether a stable polarization state is attained.

**Table 3:** Parameters used in the simulations of epithelial polarization.

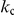	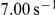	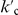	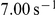	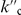	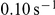	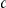	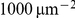
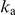	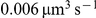	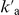	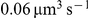	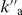	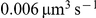	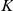	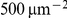
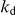	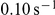	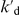	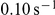	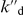	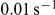	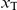	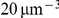
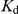	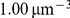	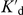	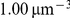	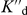	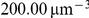	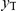	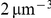

We have simulated Model (1–8) with parameter values compatible with the interactions described in Fig. 10 for the process of epithelial polarization. At initial time the plasmamembrane is in a uniform PIP3-rich state. We then create a circular patch of the PIP2-rich phase of radius 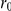 and investigate its dynamics to check whether a stable polarization state is attained.

**Figure 11:**
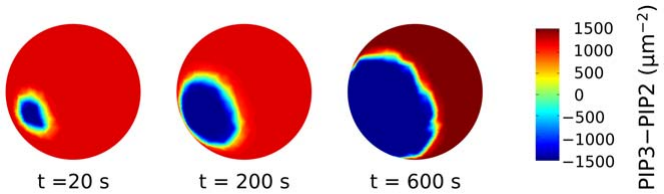
Growth of the PIP2-rich phase (blue lower patch). The color scale shows the gradation of PIP2 content: the color is the relative concentration difference between PIP3 and PIP2 at a given site. The system at initial time is in a uniform PIP3-rich phase (red), apart from an initial PIP2-rich seed germ of size larger than the threshold radius. Then, a PIP2-rich patch becomes apparent and its radius saturates to an equilibrium value.

Patches smaller than a threshold radius 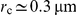 are dissolved by diffusion and thermal processes and do not impair the stability of the uniform PIP3-rich phase. Patches larger than 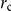 grow in time triggering the separation of the plasmamembrane surface in a PIP2-rich and a PIP3-rich region, and eventually reach an equilibrium, thus completing the separation into a PIP2-rich apical region and a PIP3-rich basolateral region (Fig. 11 and Ref. [37]). The critical radius for nucleation and the final PIP-patch size are functions of the PI3K/PTEN ratio 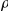 [37]. This fact suggests that the precise amount of PI3K and PTEN is a critical parameter for the establishement of epithelial polarity, providing an explanation for the experimental observation that deletion of a single PTEN allele can interfere with epithelial cell polarization and foster invasion of carcinoma cells [40].

In the process of epithelial apico-basal symmetry breaking the roles of the PIP2- and PIP3-rich phases are reversed with respect to chemotaxis. More importantly, in chemotacting cells the tendency of the cell membrane to separate in two distinct signaling domains is triggered by shallow stimulation gradients, but can also take place spontaneously [4]. On the contrary, in order to preserve well-organized geometry of epithelia, the process of apico-basal symmetry breaking must be tightly regulated and cannot take place randomly [34]. It is worth observing here that a similar network topology may induce different dynamical behaviors, depending on parameter values. The model suggests that in chemotacting cells, the high sensitivity to shallow chemoattractant gradients depends on the existence of a low potential barrier separating the PIP2- and PIP3-rich phases. On the contrary, in the case of epithelial polarization a high potential barrier prevents the random occurrence of phase separation, making it a highly controlled process. In other words, our findings suggest that in eukaryotic chemotaxis germs of the PIP3-rich phase are created in the PIP2-rich phase by a process of *homogeneous nucleation* triggered by a random fluctuation, while in epithelial polarization a single germ of the PIP2-rich phase is created in the PIP3-rich phase by an active process, *i.e.* by a process of *heterogeneous nucleation*. Notably, this prediction is in agreement with the observation that lumen formation depends on the delivery at the plasmamembrane of exocytic vesicles containing PIP2 and apical proteins [41].

### Budding yeast

Exposure to mating pheromone of haploid *Saccharomyces cerevisiae* cells results in polarized growth towards the mating partner [42]. Proteins involved in signaling, polarization, cell adhesion, and fusion are localized at the tip of the mating cell (shmoo) where fusion will eventually occur. Polarization involves localization of the small GTPase Cdc42 and of its guanine nucleotide exchange factor (GEF), Cdc24. The expression of a constitutively activated form of Cdc42 is sufficient to cause polarization in otherwise nonpolarized cells [43].

During budding, polarization is independent from extracellular cues [42]. At the G1–S phase transition of each cell cycle, yeast cells polarize to form a bud in a direction specified by a remnant from the previous round of budding, the bud scar. Haploid cells form new buds adjacent to the previous bud scar. Diploid cells form new buds alternating between both cell poles, resulting in a bipolar budding pattern.

**Figure 12:**
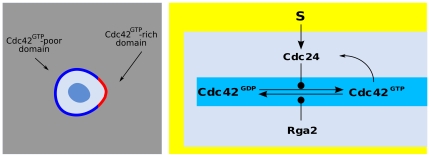
Model of cell polarization for budding yeast. With respect to the scheme in Fig. 1, we identify 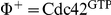, 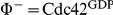, 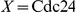, 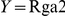.

The Cdc24 GTPase is activated by Cdc42 via the scaffold protein Bem1, resulting in the amplifying feedback loop of Fig. 12. Moreover, several GTPase activating proteins (GAP), such as Rga2, can negatively regulate Cdc42 [44]. The structure of this signaling network fits with the abstract scheme (1–8, Fig. 1) once we identify 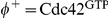, 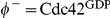, 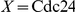, 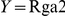.

It has been observed that intermittent, or “flickering” polarization may arise as a consequence of feedback mechanism as the one shown in Fig. 12 [45]. The model proposed in Ref. [45] is a limit case of our more general model, obtained by neglecting receptor activation and considering the limit of small number of bound Cdc42 molecules (see Supplementary Material Text S1). In this limit, 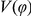 has a single potential well and no stable polarization can be observed. However, intermittent signaling patches can still arise due to the interplay of chemical and reaction noise with the nonlinear feedback. In order to study the stochastic dynamics of intermittent patches we have simulated the full stochastic model (1–8) by Gillespie's algorithm (see Methods).

**Figure 13:**
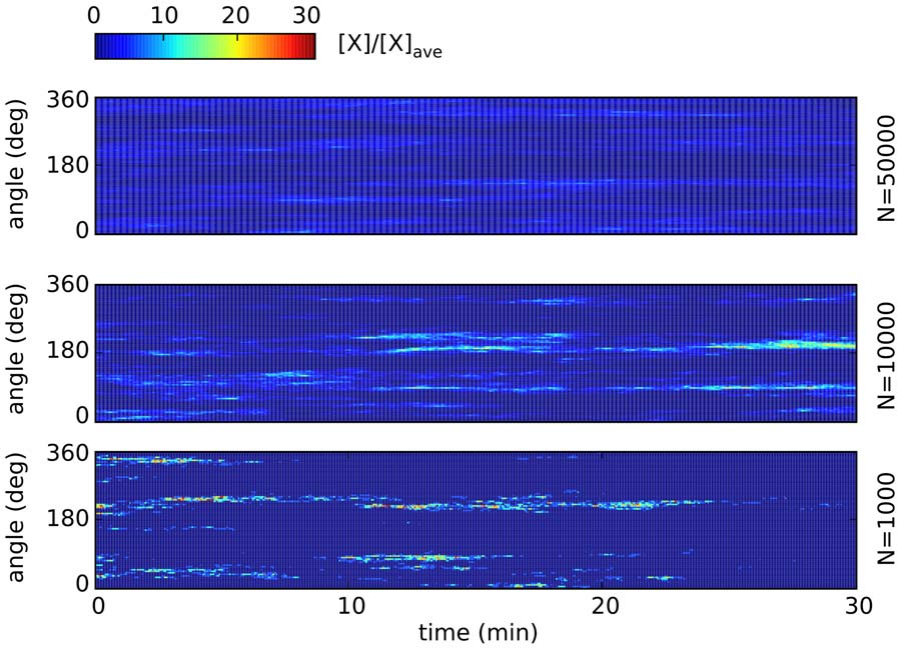
Intermittent and persistent polarization obtained by simulation of model (1–8). In the graphs we plot concentrations of membrane-bound molecules along a 1 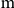 thick cross section of the plasmamembrane *vs.* time, normalized with the average membrane concentration. Upper three rows: small number 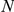 of A-molecules (PTEN in Ref. [1], or Cdc24 in Ref. [45]). Intermittent polarization as shown here was already described in [1]. The graphs of our realistic surface model are similar to those obtained in Ref. [45] in the monodimensional case. Patches of signaling molecules randomly form and disappear. Observe that patches are the macroscopic counterpart of clans of signaling molecules, as defined in [45]. Parameter values were taken as follows: diffusivity of membrane-bound molecules is 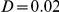, 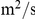, [A] = 1, 10, 50 nM, the decay rate of 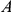 is adjusted in order to get 10% of A molecules bound to the plasmamembrane, all other parameters are as in [6], [42].

**Figure 14:**
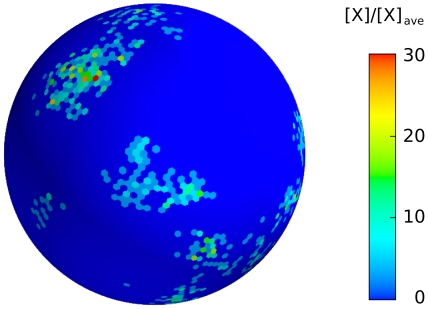
The 3D behavior of intermittent Cdc42 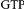
 **patches.** The graphs of our realistic surface model are similar to those obtained in the one-dimensional model of Ref. [45]. It is worth observing here that intermittent, as opposed to stable, patch formation is here a consequence of the particular, small-concentration limit considered in [45].

Fig. 13 shows the time evolution of simulated Cdc42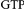 levels along a major cell cross section, while Fig. 14 shows the 3D behavior of intermittent Cdc42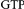 patch formation. Values of the parameters used in the simulations are shown in Table 4. The graphs of our realistic surface model are similar to those obtained in the one-dimensional model of Ref. [45]. It is worth observing here that intermittent, as opposed to stable, patch formation is here a consequence of the particular, small-concentration limit considered in [45]. However, our previous analysis shows that, if working in the appropriate parameter range, the signaling pathway described by Fig. 12 is bistable and capable of producing persistent polarized patches as those shown in Figs. 8, 9, in full agreement with experimental data [42].

**Table 4:** Parameters for simulations of budding yeast.

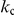	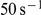	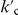	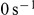	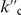	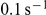	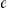	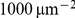
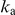	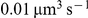	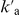	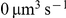	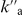	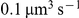	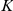	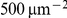
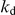	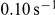	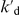	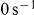	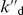	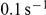	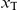	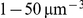
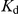	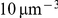	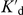	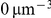	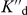	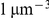	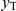	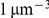

Exposure to mating pheromone of haploid *Saccharomyces cerevisiae* cells results in polarized growth towards the mating partner. Proteins involved in signaling, polarization, cell adhesion, and fusion are localized at the tip of the mating cell (shmoo) where fusion will eventually occur. Polarization involves localization of the small GTPase Cdc42 and of its guanine nucleotide exchange factor (GEF), Cdc24. The expression of a constitutively activated form of Cdc42 is sufficient to cause polarization in otherwise nonpolarized cells.

### Ras signaling domains

Ras GTPases are lipid-anchored G proteins which play a fundamental role in cell signaling processes [46]. Ras acts as a molecular switch with “on” (GTP-bound) and “off” (GDP-bound) states, the former promoting the activation of effector proteins. Ras activation is important for instance for the development of T and B lymphocytes and for their functions directed against invading pathogens [47]. Ras proteins have been observed to form dynamic non-overlapping domains (nanoclusters) in the inner leaflet of the plasmamembrane [48]–[50]. It has been shown that Ras clustering results in a prolonged immobilization at the plasmamembrane and in increased MAP-kinase activation [51].

The activation of Ras by receptor tyrosine kinases proceeds through the recruitment of the Ras-GEF Son of sevenless (Sos) to the plasma membrane [52], [53]. It was discovered recently [53] that catalysis of Ras-GDP to Ras-GTP aided by SOS is 75-fold faster when a membrane associated SOS molecule is bound to Ras-GTP at an allosteric site. This mechanism introduces positive feedback regulation of Ras activation, which in the presence of slow diffusion, may result in clustering of activated molecules on the plasma membrane [12], [48]. Moreover, several Ras-GAP proteins can negatively regulate Ras activation [54].

In Dictyostelium, Ras signaling domains have been observed at the leading edge of chemotaxing cells [25].

**Figure 15:**
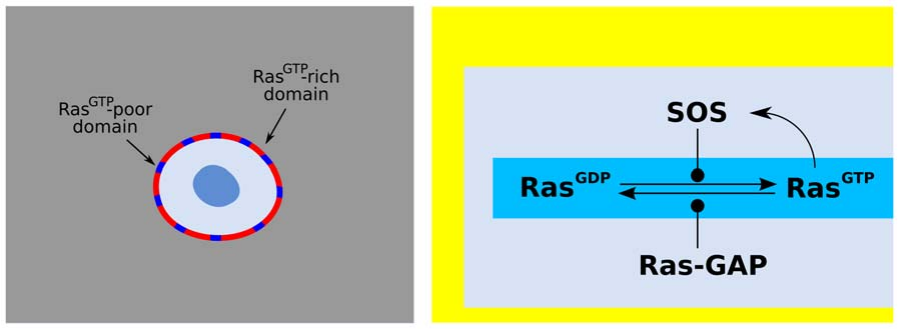
Ras activation pathway. With respect to the scheme in Fig. 1., we identify 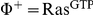, 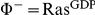, 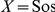, 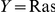-GAP.

The Ras activation pathway (Fig. 15) is still another realization of the abstract scheme described in Fig. 1, with the identification 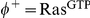, 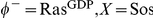, 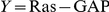. In particular, the creation of germs of the 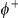-rich phase is expected to take place via the formation of small germs of the new phase by the action of random thermal and chemical fluctuations, as observed in [12]. Our previous analysis shows that the Ras-GDP/Ras-GTP system can support the formation of both intermittent nanoclusters, or stable signaling domains of Ras-GTP, depending on parameter values. The formation of patches of the 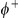-rich phase is expected to be intermittent outside of the bistable region II,III in Fig. 3, and generating stable signaling domains in the interior of these regions. Moreover, we expect that the role of the finite cytosolic reservoir of SOS should be central in tuning the cell plasma membrane towards coexistence of the 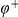-rich and 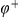-poor phases.

## Discussion

Generation of spatio-temporally localized signaling domains is an ubiquitous feature of many cellular functions, such as chemotaxis, epithelial morphogenesis and mating. Interestingly, the organization of most of the corresponding molecular machineries involves molecules that exist in two alternative biochemical states, phosphatydilinositol and GTPases being prominent examples. The transition between these two states is typically controlled by the activity of a couple of counteracting enzymes. In addition, substrate-to-enzyme feedbacks can often generate hypersensitive responses. This ubiquitous pathway architecture can be formally described as a Goldbeter-Koshland hypersensitive module coupled with one or more reinforcing feedback loops. Here we have presented a general mathematical analysis of its properties.

Hypersensitivity has been been considered for a long time as a way for a biochemical system to realize abrupt step responses to small variations in input concentrations. In the usual treatment, uniform spatial concentrations are considered. Here we have extended this approach to the case of spatially distributed, diffusive systems with reinforcing feedback loops. Our results show that in this context, Goldbeter-Koshland hypersensitivity can induce the separation of a biological system such as the cell plasmamembrane into distinct signaling domains. This simple principle appears of sufficient generality to explain the emergence of polarized domains in several basic biological settings, such as differentiation, proliferation, migration, and morphogenesis.

The idea that chemotactic cell polarization may result from the simple ingredients of bistability induced by a positive local feedback loop in a signaling network and global control induced by shuttling of enzymes between the cytosol and the membrane was advanced in our previous works [1], [2], [55]. Other authors have adopted similar models [11], [13], [56]–[60]. Alternative models include “local excitation – global inhibition” [24], [61], [62], Turing-like [65] and excitable systems [66]. An extensive review of cell polarization models can be found in [63], [64].

It is worth observing here that the coupling of local bistability and diffusion is an alternative way to produce patterning in an extended system, with respect to the better known mechanism of Turing instabilities [65], [67], [68]. The main difference is that Turing instabilities are linear instabilities of the uniform (mixed-phase) state. Instead, in our model an “energy” barrier has to be overcome to pass from the uniform state to the phase-separated state, similarly to what happens in the theory of phase separation in statistical physics. The mean-field, uniform state is stable, but a finite perturbation may break it. The finite perturbation may be produced either by random fluctuations (noise) in the system, or by an external perturbation, such as the introduction of a finite-size germ of one of the two phases. This way, the process of phase separation may be finely controlled by the signaling network.

Feedback loops participating in cell polarization may involve the actin cytoskeleton [24], [30]–[32], [69]–[71]. Such actin-mediated feedbacks may imply the active transport of signaling molecules along cytoskeleton filaments [69]–[71]. As long as the local geometry of actin filaments is neglected, active transport along cytoskeleton filaments may be taken into account in our model through renormalized values of the adhesion rates 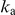 (compare, e.g., Eq.s. 1–8, or Eq.s B.1–B.5 from Ref. [3], with Eq.s 1–4 from Ref. [71]). Different local geometries (e.g., astral or radial) of actin filaments in the proximity of the cell membrane may however facilitate or inhibit the development of instabilities leading to cell polarization [71], [72]. These effects are expected to be particularly relevant if polarization is driven by Turing-like instabilities. In our bistable scenario, the effect of local inhomogeneities in the distribution of signaling molecules in the proximity of the cell membrane has still to be investigated.

It has been recentely shown that a bistable scenario for diffusible signaling molecules satisfactorily accounts for the polarization of fertilized oocytes, and for the flow of actomyosin cell cortex that is observed in the process, provided that the polarization pathway is properly coupled to the mechanics of membrane advection [73].

It is important to notice that a definitive assessment of the roles of Turing vs. bistable mechanisms in cell polarity can not be done by purely theoretical means. For instance, the mathematical description of the same signalling pathway may involve either nonlinearities leading to bistable behavior, if Michaelis-Menten saturation terms are taken into account to describe enzymatic kinetics (as we did here), or to Turing-like systems [65] if reactions are believed to be working in the non-saturating regime. Therefore, the Turing vs. bistability alternative can be ultimately solved only by performing accurate, targeted experiments.

Our model is simple enough to be studied by analytical methods, that in particular allow to derive a phase diagram showing the region of parameters where the coexistence of two signaling plasmamembrane domains is allowed. The dynamics leading to cell polarization can then be studied by introducing an *effective energy function* which encodes many of the qualitative and quantitative properties of the real process. This fact allows to draw a useful analogy with physical processes, such as the formation of precipitate from a supersaturated solution, and to take advantage of a well-developed mathematical theory of their dynamical properties. The main feature emerging from this analysis is that the system dynamics depends only on robust properties of the pathway architecture, such as bistability and self-tuning, and not on the precise values of microscopic details such as diffusion and chemical rate constants, or the identity of individual biochemical factors. This unified picture suggests that polarization phenomena observed in apparently distant biological models are sharing a set of common features.

Our theoretical framework leads to well-defined predictions about the polarized response of eukaryotic cells under both uniform and gradient stimulation conditions. To validate these predictions it would be necessary to systematically collect time-lapse, 3D microscopy data of signaling patches induced by controlled space-time extracellular stimulation patterns, such as those that can be realized by computer-controlled microfluidics.

Such measurements should be performed also by modulating the cellular levels of 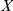 and 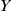 enzymes, e.g. by plasmid or virus-mediated overexpression as well as by gene silencing. Our theory suggests that when a cell is uniformly stimulated, the dynamic of signaling domains should show similar features in different biological models: at appropriate stimulation levels, signaling domains should appear as small intermittent spots that coarsen in time in a process where larger domains grow at the expense of smaller ones, finally reaching a configuration characterized by a single polarized cap.

A threshold in the stimulation levels is expected to separate a dynamics characterized by a “sea” of intermittent, small signaling domains (below threshold) and the above-mentioned coarsening dynamics leading to a single polarized cap (for above threshold stimulation levels). It is worth observing here that at low stimulation levels the signaling mechanism can be influenced by autocrine stimulation loops, which must therefore be accurately monitored.

The dynamics of signaling patches under gradient stimulation conditions is predicted to be quite similar to the dynamics observed under uniform stimulation conditions, except that polarization times should be much shorter and the direction of polarization should be aligned with the direction of the stimulation gradient.

## Methods

The diffusion on the plasmamembrane has been simulated with a Finite Element Method for the Laplace-Beltrami operator and a suitable discretization of the spherical surface. The resulting ODE system has been approximated by using a Runge-Kutta stiff solver [74].

In the simulation of eukaryotic chemotaxis, noise intrinsic in the reaction-diffusion system is taken into account by adding an additive Poissonian random perturbation in the r.h.s. of (1, 2). In detail, nodes in the lattice are chosen randomly with rate 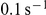 in time and with uniform probability in space and their state is set to 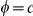.

Reaction-diffusion kinetics has been simulated using Gillespie's method [75], [76]. At time zero, a random number is generated to determine the next reaction or elementary diffusion process to occur, with a probability proportional to the corresponding 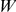 factor from Table 1. Then, the time is advanced as a Poissonian process with a rate again determined by the 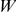 factors. These steps are repeated iteratively until the desired simulation time is reached.

## Supporting Information

File S1 Supplementary material.
(PDF)
