# Supplementary material for: A Bistable Model of Cell Polarity
Source: PLoS One. 2012 Feb 23;7(2):e30977. doi: 10.1371/journal.pone.0030977 (PMC3285628; doi:10.1371/journal.pone.0030977)
Supplement: File S1 — Supplementary material. (PDF) [file pone.0030977.s001.pdf]

Supplementary Material

A bistable model of cell polarity

Matteo Semplice[1], Andrea Veglio[2], Giovanni Naldi[3,*], Guido Serini[4], Andrea Gamba[5,6,7]
1 Department of Physics and Mathematics, Università dell'Insubria, Como, Italy
2 Genomes and Genetics Department, Unit Physics of Biological Systems, Institut Pasteur, Paris, France
3 Department of Mathematics "F. Enriques", Università degli studi di Milano, Milano, Italy
4 Laboratory of Cell Adhesion Dynamics, Institute for Cancer Research and Treatment and Department of Oncological Sciences, Università degli studi di Torino, School of Medicine, Candiolo, Italy.
5 Department of Mathematics, Politecnico di Torino, Torino, Italy
6 Laboratory of Systems Biology, Institute for Cancer Research and Treatment, Università degli studi di Torino, School of Medicine, Candiolo, Italy.
7 INFN, Torino, Italy,
$\ast$ E-mail: giovanni.naldi@unimi.it

Stable phases

We start by looking for solutions of equation (12) in the main text, corresponding to a uniform chemical phase. They are obtained from the condition

$$V'(\phi) \quad = \quad 0, \tag{1}$$

which, once solved, gives two stable equilibrium values $\varphi_+ = c,\ \varphi_-$ and one unstable equilibrium $\varphi_{\rm unst}$. The φ_+, φ_- values correspond to distinct, stable, uniform chemical phases, enriched respectively in the signaling molecules Φ^+ and Φ^-. We refer to the existence of two distinct stable chemical phases as *bistability*. The explicit concentration values are

$$\varphi_{unst}, \varphi_- \quad = \quad \frac{1}{2}\left[-(S+T+c) \pm \sqrt{(S+T-c)^2 - 8KS}\right] \tag{2}$$

where

$$S \quad = \quad \frac{c\rho\sigma}{\rho+1}, \qquad T = \frac{\rho-1}{\rho+1}(2\kappa+1)c, \qquad \kappa = \frac{K}{c}, \tag{3}$$

$$\rho \quad = \quad \left(\frac{k_c'' k_a'' k_d}{k_c k_a k_d''}\right)\frac{X_f}{Y_f}, \quad \sigma = 2\left(\frac{k_c' k_a' k_d''}{k_c'' k_a''^{*} k_d'}\right)\frac{s}{c}. \tag{4}$$

Eqs. (2-4) show that the concentration values (2) are completely controlled by the *enzyme ratio* ρ, which measures the relative strength of the counteracting X and Y enzimes, and by the *renormalized activation signal* σ.

A graph of the concentration values in the two stable phases is given in Figure 2 of the main text. An important consequence of the existence of two distinct, locally stable phases is that different regions of the cell membrane can be occupied by different phases, giving rise to patterning into distinct *signaling domains*.

The $\varphi_\pm, \varphi_u$ values have actual meaning only if they correspond to non-negative concentrations, *i.e.* if they satisfy the inequality:

$$-c \quad < \quad \varphi_- < \varphi_u < \varphi_+ < c \tag{5}$$

which is equivalent to the following condition on the enzyme ratio ρ and saturation constant κ:

$$\rho_{\min} \quad < \quad \rho < \rho_{\max}, \qquad \kappa < \kappa_{\max} \tag{6}$$

with

$$\rho_{\max} \quad = \quad \frac{4\kappa(1+\kappa) + (2+6\kappa)\sigma - 4\sqrt{(2\kappa + 6\kappa^2 + 4\kappa^3)\sigma + (\kappa + 2\kappa^2)\sigma^2}}{(2\kappa - \sigma)^2} \tag{7}$$

$$\rho_{\min} \quad = \quad \frac{2\kappa}{(2+\sigma)(1+\kappa)} \tag{8}$$

$$\kappa_{\max} \quad = \quad \frac{1}{2}\left[\left(1 + \frac{4}{\sigma}\right) + \sqrt{5 + \frac{16}{\sigma} + \frac{16}{\sigma^2}}\right] \tag{9}$$

These formula define the *bistability region* shown in Figure 3 of the main text.

In the limit of negligible feedback $k_c'' k_a'' / k_d'' \to 0$ the conditions (6) reduce to the result in [3], see main references.

Coexistence line

The cell membrane is *polarized* when it divides into two complementary regions, stably occupied by one of two distinct chemical phases, and separated by a thin diffusive interface. Stable polarized equilibria are reached when the effective energy $\mathcal{F}$ is minimal, *i.e.* when both terms in (15) take on their minimal values. If *e.g.* $V(\varphi_+) < V(\varphi_-)$, no polarized configuration can be stable because the energy can still decrease by extending the area covered by the φ_+ phase, which has lower energy than the φ_- phase. The same is true if $V(\varphi_+) > V(\varphi_-)$. Therefore, stability of polarized equilibria (or *phase coexistence*) is possible only if the following mathematical condition is satisfied:

$$\int_{\varphi_-}^{\varphi_+} V'[\phi] \mathrm{d}\phi \quad = \quad 0$$

that can be set in the form of an implicit integral equation for the value at equilibrium of the enzyme ratio ρ_{eq}:

$$\rho_{\mathrm{eq}} \quad = \quad \left[\int_{\varphi_-(\rho_{\mathrm{eq}})/c}^{\varphi_+(\rho_{\mathrm{eq}})/c} \frac{(1-\phi)(1+\phi+\sigma)}{(2\kappa+1-\phi)} \mathrm{d}\phi\right]^{-1} \cdot \int_{\varphi_-(\rho_{\mathrm{eq}})/c}^{\varphi_+(\rho_{\mathrm{eq}})/c} \frac{1-\phi^2}{2\kappa+1+\phi} \mathrm{d}\phi$$

This equation can be numerically solved to determine the *phase coexistence line* (Figure 3) where stable polarized configurations are possible. For $\kappa = 1$ an approximate expression for the coexistence line, valid for both small and large σ, is

$$\rho_{\mathrm{eq}} \quad = \quad \frac{1}{1+\sigma}$$

Patch area

In the equilibrium state characterized by the equilibrium value ρ_{eq} of the enzyme ratio (19), the A_+ and A_- areas of the circular caps occupied respectively by the φ_+ and φ_- phases are determined by the integral constraints. The A_+ and A_- areas can be explicitly computed if the area of the interfacial region separating the two caps is negligible with respect to the cell membrane area A, so that *e.g.* $\overline{\phi}_+ \simeq \varphi_+ A_+ / A$:

$$A_- \quad = \quad \frac{\left(1 + \frac{k_c''}{k_c'}\frac{\sigma}{2}\right)\frac{\rho_{\mathrm{eq}}}{\rho_T} + \frac{k_d''/k_a''}{c\theta}\left(\frac{\rho_{\mathrm{eq}}}{\rho_T} - 1\right)}{\frac{c-\varphi_-}{2c} + \frac{k_d''/k_a''}{k_d/k_a}\frac{\rho_{\mathrm{eq}}}{\rho_T}} A, \quad A_+ = A - A_-, \qquad \rho_T = \left(\frac{k_c'' k_a'' k_d}{k_c k_a k_d''}\right)\frac{X_T}{Y_T}$$

Small concentration limit

In [39] it was observed that intermittent, or "flickering" polarization may arise as a consequence of feedback mechanism like that of Figure 12, and a computational model was proposed for that behavior and the formation of spatiotemporal structure was simulated using that model. The model proposed in [39] is a limit case of our general model, obtained by neglecting receptor activation ($k'_c k'_a / k'_d = 0$) and considering the limit of *small number of bound Cdc42 molecules* ($\phi^+ \ll c$, $\phi^- \approx c$). In this limit, $V(\varphi)$ has a single potential well and no stable polarization can be observed. However, intermittent signaling patches can still arise due to the interplay of chemical and reaction noise with the nonlinear feedback.

If in Model (1-8) we identify $\phi^+ = \mathrm{Cdc}\,42$, $X = \mathrm{Cdc}\,24$, in the $\phi^+ \ll c$ limit we get

$$\begin{aligned}\frac{\partial u}{\partial t} &= D\Delta u + k_{\mathrm{on}}(1-h) + k_{\mathrm{fb}}(1-h)u - k_{\mathrm{off}}u = V'(\varphi)\\ \frac{\mathrm{d}h}{\mathrm{d}t} &= k_{\mathrm{on}}(1-h) + k_{\mathrm{fb}}(1-h)h - k_{\mathrm{off}}h\end{aligned}$$

where for ease of comparison the notations from [39] are used:

$$u = \phi^+, \quad 1-h = \frac{X_f}{X_T}, \quad k_{\mathrm{fb}} = \frac{X_T c}{K'' + c}\frac{k''_c k''_a}{k''_d}, \quad k_{\mathrm{on}} = \frac{Y_f c}{K}\frac{k_c k_a}{k_d}, \quad k_{\mathrm{off}} = \frac{Y_T}{K}k_c$$

With the values from Table 4 we get

$$k_{\mathrm{fb}} = 0.1\,\mathrm{s}^{-1}, \qquad k_{\mathrm{on}} = 10^{-4}\,\mathrm{s}^{-1}, \qquad k_{\mathrm{off}} = 0.1\,\mathrm{s}^{-1}$$

corresponding to the values from [39].
